# Parkinson’s Disease Recognition Using Decorrelated Convolutional Neural Networks: Addressing Imbalance and Scanner Bias in rs-fMRI Data

**DOI:** 10.3390/bios14050259

**Published:** 2024-05-19

**Authors:** Pranita Patil, W. Randolph Ford

**Affiliations:** Department of Analytics, Harrisburg University of Science and Technology, Harrisburg, PA 17101, USA; rford@harrisburgu.edu

**Keywords:** class bias, DcCNN, decorrelation, deep learning, FE-DcCNN, invariant features, Parkinson’s disease, rs-fMRI image, scanner bias

## Abstract

Parkinson’s disease (PD) is a neurodegenerative and progressive disease that impacts the nerve cells in the brain and varies from person to person. The exact cause of PD is still unknown, and the diagnosis of PD does not include a specific objective test with certainty. Although deep learning has made great progress in medical neuroimaging analysis, these methods are very susceptible to biases present in neuroimaging datasets. An innovative decorrelated deep learning technique is introduced to mitigate class bias and scanner bias while simultaneously focusing on finding distinguishing characteristics in resting-state functional MRI (rs-fMRI) data, which assists in recognizing PD with good accuracy. The decorrelation function reduces the nonlinear correlation between features and bias in order to learn bias-invariant features. The publicly available Parkinson’s Progression Markers Initiative (PPMI) dataset, referred to as a single-scanner imbalanced dataset in this study, was used to validate our method. The imbalanced dataset problem affects the performance of the deep learning framework by overfitting to the majority class. To resolve this problem, we propose a new decorrelated convolutional neural network (DcCNN) framework by applying decorrelation-based optimization to convolutional neural networks (CNNs). An analysis of evaluation metrics comparisons shows that integrating the decorrelation function boosts the performance of PD recognition by removing class bias. Specifically, our DcCNN models perform significantly better than existing traditional approaches to tackle the imbalance problem. Finally, the same framework can be extended to create scanner-invariant features without significantly impacting the performance of a model. The obtained dataset is a multiscanner dataset, which leads to scanner bias due to the differences in acquisition protocols and scanners. The multiscanner dataset is a combination of two publicly available datasets, namely, PPMI and FTLDNI—the frontotemporal lobar degeneration neuroimaging initiative (NIFD) dataset. The results of t-distributed stochastic neighbor embedding (t-SNE) and scanner classification accuracy of our proposed feature extraction–DcCNN (FE-DcCNN) model validated the effective removal of scanner bias. Our method achieves an average accuracy of 77.80% on a multiscanner dataset for differentiating PD from a healthy control, which is superior to the DcCNN model trained on a single-scanner imbalanced dataset.

## 1. Introduction

Parkinson’s disease (PD) [1] is characterized by a lack of dopamine transmitters due to the degeneration of melanin cells in the pars compacta (posterior part) of the substantia nigra, and PD patients show several cognitive deficits which include executive functioning, visuospatial abilities, and memory loss. The symptoms of PD include shaking, slow movements, walking problems, behavioral problems, speech problems, etc. Diagnosis of PD generally includes assessment of behavior, neuroimaging, physical examination, biological sampling, and clinical data. The false-positive rate for PD is higher in the early stage and high at the final diagnostic stage. In the past few years, studies in neuroimaging modalities have provided more profound and valuable insights into the underlying mechanism of PD.

Parkinson’s disease remains the second most common neurodegenerative disorder. However, still, there is an unknown factor in the cause of PD, which makes PD a very important area of study. Motor symptoms, along with cognitive impairment, are also found as common disabling symptoms in PD. The mechanism underlying cognitive dysfunction in PD remains ambiguous, unlike that of motor symptoms. Many studies have been conducted on PD using clinical and biomarker data. Most of them are driven by hypotheses and handcrafted feature extraction methods which are based on pathology-related background knowledge. Recently, neuroimaging has been considered an important information source for neurodegenerative disease. Hence, it has also raised considerable interest from the PD community. Diagnosing Parkinson’s disease based on diagnostic tests and radiologists’ reading on neuroimages is oftentimes prone to mistakes. So, there is a gray area in the PD diagnosing research field where the unknown cause of PD, no precise test for PD, and a high misdiagnosis rate are present. There is a need for highly accurate and reliable results. This research may be of use to the medical community in a screening setting, to understand how and why PD develops, and to search for solutions to stop or avoid the progression of the disease.

Currently, no specific test exists to diagnose Parkinson’s disease. There are a few diagnostic tests that physicians use to diagnose Parkinson’s disease based on medical history, review of signs and symptoms, physical examination, blood test, and neuroimaging tests. As PD progresses, it becomes harder to prevent or slow the changes through medication. For this reason, in 2016, experts developed new criteria [2]. These include three steps. The first step includes assessing the probability based on the age that the diagnosis will be PD. In the second step, physicians assess information based on variables such as whether the person is male or female, environmental risks, caffeine use and smoking, genetic factors, family history, or genetic tests. Sometimes findings based on these results of scans and other diagnostic tests show early signs and symptoms, which include constipation, loss of the sense of smell, and difficulty with movement. The third and final step consists of calculating the outcome by multiplying all the factors together and then comparing this total likelihood ratio with a threshold measure. If the comparison indicates a total likelihood ratio higher than 80 percent that PD is present, the physician will diagnose that patient with the early stages of PD. Most commonly, a patient with a 75–80 percent total likelihood will have symptoms that may or may not relate to PD, e.g., constipation and depression, whereas a patient with a 95–97 percent total likelihood will have symptoms that are closely related to PD, e.g., rapid eye movement (REM) sleep behavior disorder, where a person experiences sudden and rapid movements and vocalizations during vivid dreams.

Deaths caused by PD have increased significantly over the years. The diagnosis of PD used in hospitals relies mainly on a combination of different diagnostic tests and symptom assessments. It is still difficult to make an accurate prediction of PD. Neuroimaging data such as magnetic resonance imaging (MRI), resting-state functional magnetic resonance imaging (rs-fMRI), single-photon emission tomography (SPECT), dopamine transporter imaging (DAT), 123I-ioflupane-SPECT (DaTscan), diffusion tensor imaging (DTI), positron emission tomography (PET), and computed tomography (CT) scans can be used to diagnose PD. However, CT scans and MRI images sometimes do not show patterns in images to distinguish PD from a healthy patient. In contrast, SPECT is a commonly used method but suffers from high cost and time issues and requires the injection of radioactive material. Radiologists generally use one of these neuroimages to diagnose PD, but the methods are proven to be more prone to mistakes. Recent research and studies [3,4] have shown that DTI and rs-fMRI can be used to predict PD and are found to be promising methods for the diagnosis of PD. However, in order to capture DTI images, the patient has to remain still for a longer period, i.e., half an hour. Since DTI is a relatively new technique, it is difficult to find hospitals equipped with DTI scanners.

Current existing methodologies such as [5,6] do not use rs-fMRI using decorrelated CNN to detect PD. Therefore, the processing of rs-fMRI with single-scanner and multiscanner settings using decorrelated CNN techniques to diagnose PD is not yet explored. This novel research study evaluates the prediction of PD on noninvasive and comparatively less expensive neuroimaging data such as rs-fMRI in single-scanner and multiscanner settings using models that use a decorrelated convolutional neural network. Since available neuroimaging data are limited and the majority of the data are class-imbalanced, this study also provides a novel decorrelation-based deep learning fusion approach to mitigate class bias. Further, we also explore the use of multiscanner rs-fMRI data, which are obtained by combining different datasets from different scanners, not only to balance the dataset but also to increase the size of the dataset and to improve the performance of the models. However, this leads to an undesirable increase in variance caused by scanner and acquisition protocol differences, including scanner upgrade, scanner drift, and gradient nonlinearities. The same framework of decorrelation-based deep learning is used to produce features that are invariant to scanner and acquisition protocols while still being capable of not impacting the performance of the PD recognition task. The main objective of our proposed model is to recognize Parkinson’s disease while addressing scanner- and class-bias issues found in neuroimaging data.

Our main contributions in this study are as follows:

- The introduction of a decorrelation function to mitigate the biases found in neuroimaging datasets while focusing on the models’ performance;
- The use of a decorrelated CNN and convolutional gated recurrent unit–convolutional neural network for the first time to detect PD but also to address class-imbalance and scanner-bias issues found in single-scanner and multiscanner settings, respectively;
- A comparison of our proposed models with the baseline models in terms of bias mitigation and accuracy.

The rest of this paper is structured as follows: Section 2 briefly reviews the related work; Section 3 provides a brief description of the proposed methodologies, involved PD datasets, and preprocessing techniques; Section 4 reports the results and comparison with existing methodologies; and Section 5 discusses the performance of our proposed method for PD detection. Lastly, Section 6 concludes the research and provides opportunities for future work.

## 2. Prior Work

Two centuries ago, James Parkinson presented the first medical description of Parkinson’s disease in 1817. Today, Parkinson’s disease is the second most common neurodegenerative disorder. The pathophysiology of Parkinson’s disease (PD) is the study of the functional processes that occur in PD, which is only partially understood. Currently, what we know about PD is that the loss of neurons in the substantia nigra pars compacta part of the brain and the presence of Lewy bodies leads to the loss of dopamine (a neurotransmitter). This damaged neurotransmitter ultimately prevents normal function in the basal ganglia, which causes the motor symptoms of PD and cognitive impairment. Common motor symptoms observed in PD include tremors, slowness, stiffness, rigidity, swallowing problems, balance problems, unpredictable movements, difficulty initiating or controlling movement, cramping, and speech problems. Cognitive issues, such as short-term memory loss, difficulty following complicated instructions, or a loss of multitasking ability, may also occur in PD patients. Some people will have several symptoms, whereas others will have only a few. It has been observed that deaths caused by PD have increased significantly over the years. This is mainly because PD is difficult to diagnose and can be caused by a combination of environmental, genetic, or lifestyle factors. Male gender, gait disorder, and absent rest tremor are generally associated with poorer long-term survival. According to NIH, approximately 50,000 to 60,000 Americans are diagnosed with PD each year. Because of a lack of knowledge regarding which symptoms develop and how severely and quickly symptoms develop, and since the symptoms of Parkinson’s vary from patient to patient and often overlap with other medical conditions, PD is misdiagnosed up to 30 percent of the time. It has been observed that misdiagnosis of PD is very common. So, there is a need for an automated diagnostic tool.

### 2.1. Pathology-Driven Hypotheses

In the past few years, several studies have been performed to explore the connection between clinical, biological, and imaging data to achieve an accurate diagnosis and early detection of PD. Most of these studies are driven by pathology or the underlying biology of PD and use hypotheses. According to [7,8], the α-Synuclein protein, which is a major component of Lewy pathology, accumulates and originates from cells in the gut and transmits to the brain via a vagus nerve in the patient with Parkinson’s disease. The authors performed the study on a mouse model and supported the Braak hypothesis. This research might help to prevent or halt PD progression by blocking the vagal transmission pathway in an early stage. From a genetic contribution point of view, a paper published by [9] suggests that protein products of genes help to identify the functionality of PD, whereas [10] investigated the use of α-Synuclein protein as a biomarker for PD using hypothesis testing with around 85% specificity and 52% sensitivity. In [11], the paper, using an innovative approach, suggested the use of sebum to diagnose PD, since a change in skin microflora and skin physiology can cause a change in odor in PD patients. The results (AUC 78%) to support this theory were achieved by collecting sebum samples from the participants’ upper back, using a combination of data processing techniques, such as olfactogram and chromatogram, and performing partial least-squares-discriminant analysis on these preprocessed data. The main limitation of the study is its smaller sample size. There are quite a few studies conducted to diagnose PD by using neuroimaging and clinical data.

Several papers such as [12,13,14,15,16,17] have suggested that the use of DTI metrics can provide distinguishing features to detect PD or be used as imaging biomarkers for PD. In recent years, there have been studies [18,19] in rs-fMRI, which is a fast-developing research field and helps in revealing cognitive dysfunction or increasing motor connectivity for early PD detection. All these studies perform hypothesis testing such as t-test, two-way mixed model ANOVA, comprehensive meta-analysis, etc., to find significant group differences between PD and control healthy groups. The cross-sectional study [20] claimed that serotonergic pathology plays a vital early role in the progression of PD. The study provided evidence that loss in serotonin function is observed in the very early stages of PD by using PET and SPECT scans. To assess molecular, clinical, and structural pathology, PET imaging was used. ANOVA and t-test were used for comparisons between the groups and suggested that serotonergic malfunction precedes the development of other PD symptoms, such as motor, and is related to the dopaminergic deficit by using the Braak staging scheme.

### 2.2. Data-Driven Models

Data-driven approaches, such as deep learning and machine learning, are different than conventional statistical analyses. DaTscan SPECT image analysis with a one-layer artificial neural network was developed to classify PD versus healthy patients with around 94% accuracy [21]. Machine learning-based approaches such as a support vector machine [22], a Naive Bayes classifier [23], and a boosted logistic regression model [5] were also used for PD classification using rs-fMRI data, but these were tested on very small datasets.

To overcome the drawback of feature engineering or handcrafted features, a few deep learning techniques have been deployed in the past decade. Ref. [24] used SPECT data to detect PD over control using deep 3D CNN architecture, which achieved around 96%, far higher than human evaluation accuracy, and could be used for the SWEDD group. Another study in deep learning was carried out by [25] using graph convolutional deep networks (GCNs) to fuse multiple modalities of MRI and DTI to detect PD cases and achieved around 95% AUC. In the study, a brain geometry graph (BGG) was obtained from the region of interest of MRI and brain connectivity graphs (BCGs) from the tractography of DTI and used as input to a GCN to explore spatial and frequency spectrum information. Laplacian and Fourier transform-based graph convolutions were performed on BGGs and BCGs, and then multiview pooling was performed to aggregate multiview outputs of GCNs together. The authors also used pairwise matching between outputs of multiview GCNs to increase the amount of data. In the final step, a fully connected softmax network was used for classification by using pairwise matching layer output. Ref. [26] performed PD diagnosis using a 3D convolutional neural network(3D CNN) deep learning framework on 3D MRI and patient personal information such as age and gender. The work is primarily compared with the work of [27,28] for performance comparison. The main goal of the pilot study was to integrate feature extraction and model learning into one framework to improve performance. Skull stripping by using the brain extraction technique (BET) with statistical parametric mapping (SPM) algorithms was used to remove noncerebral tissue in order to improve the speed and accuracy of the study. Flipping of the right and left hemispheres was performed in the data augmentation process. In their study, the authors claimed that using age alone in logistic regression to predict PD achieved 72% accuracy. The authors also performed image occlusion analysis to study important parts of the brain in PD diagnosis and suggested those parts are the basal ganglia and substantia nigra, along with the superior parietal part on the right hemisphere of the brain. Their proposed approach achieved 100% accuracy in distinguishing PD from healthy patients. The limitation of the study is that methodology was tested on a small-sample-size dataset.

In the [18] paper, the authors suggested that rs-fMRI can differentiate patients with early PD from healthy controls. Their study primarily consisted of calculating connectivity scores based on three regions of interest such as the caudate, putamen, and pallidum. The paper also recommended the use of rs-fMRI as a biomarker for early PD detection. Recently, rs-fMRI data were used in the early diagnosis of PD using a long short-term memory (LSTM) model by [6]. This model achieved around 72% accuracy with a small-sized dataset consisting of only 84 subjects. All the above studies were performed using identical data acquisition conditions and on a single scanner at the same site. However, larger multiscanner and multisite data are required to achieve higher generalization by building a more robust model. There are a few multisite research studies [29,30,31] which are based on fMRI. These studies focused on controlling scanner variations, but they were performed using very small datasets and were not for PD diagnosis. In addition to these studies, the ComBat harmonization approach [32] was also used for fMRI-derived connectivity measures in a multisite study but can be used only on image-derived values and predefined relationships. Deep learning methods with an attention-based channel are used on large multisite resting-state fMRI datasets without explicitly applying any scanner-bias mitigation method [33] to generalize models to multisite datasets. The federated learning approach [34] with two domain adaptation techniques, such as a mixture of expert domain adaptation to reduce the effect of a new domain on the global model and adversarial domain alignment to reduce the discrepancy between the source and target domains, is used to resolve the domain-shift issue observed in multisite fMRI datasets.

There have been many methods proposed for classifying PD using machine learning and deep learning. However, class imbalance and scanner bias remain issues in PD classification. Moreover, a minimal amount of previous research has used rs-fMRI to classify PD based on data-driven models. To the best of our knowledge, the proposed approach is the first to use a decorrelated convolutional neural network and convolutional gated recurrent unit–convolutional neural network (ConvGRU-CNN) to identify Parkinson’s disease using resting-state functional magnetic resonance imaging (rs-fMRI) data and patient information such as age and gender. Furthermore, a simple and effective distance correlation technique was used for the first time to address class-imbalance and scanner-bias issues in neuroimaging data, which allows us to generalize the proposed models to larger multisite and multiscanner settings.

## 3. Materials and Methods

Deep learning techniques in the medical domain have received increasing interest due to their ability to accurately performing tasks and extract meaningful features in neuroimaging datasets. However, the performance of deep learning models is impacted by imbalanced and multiscanner dataset issues. Imbalanced datasets exhibit skewed class distributions, while multiscanner datasets exhibit data bias or confounding effects due to variance caused by differences in scanner and acquisition protocols. In this study, we aim to resolve two issues associated with rs-fMRI datasets of PD:

1. The dataset is highly imbalanced, which introduces a class-bias issue. Hence, deep learning models trained on this dataset are biased towards the majority class. In our study, the majority class is PD patients.
2. In order to improve the performance of deep learning, the datasets from two different scanners and different studies and sites are combined. However, this leads to scanner-variant features, and hence, model predictions are dependent on a scanner.

**Figure 1:**
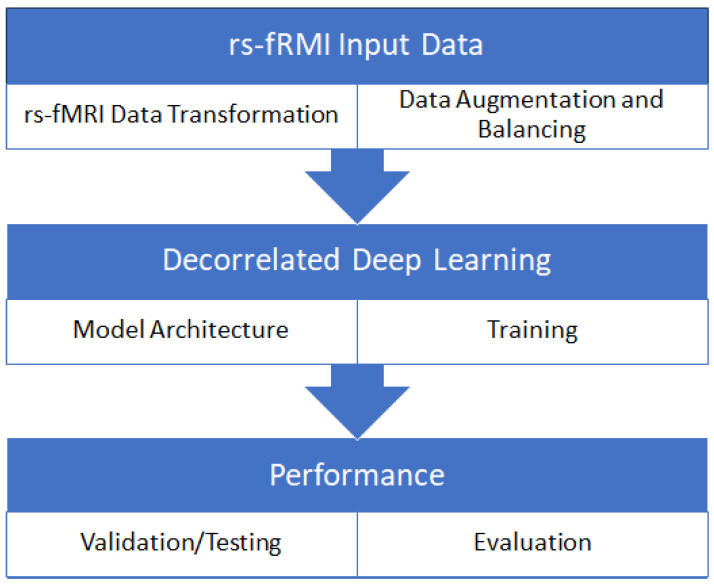
General framework of the proposed method for classification of Parkinson’s disease.

Our proposed method focuses on using distance correlation in the objective function to mitigate bias toward the majority class and scanner dependencies from features learned by deep learning. In this method, we improve the classification performance on the imbalanced dataset by decorrelating class bias from features learned by the model. Scanner dependencies in model performance are mitigated by decorrelating scanner configuration information from learned features to create scanner-invariant features. The proposed method is simple yet more effective and can be applied to the mitigation of a wide range of data-bias, confounder, class-bias, or a combination of all bias issues, as shown in our previous work [35]. The proposed DcCNN framework in this study, on the other hand, is specifically designed to address scanner-dependency and imbalance issues that are common in large clinical trials involving neuroimaging data. The proposed DcCNN model framework is shown in Figure 1. The framework mainly consists of three steps: data preprocessing, balancing the dataset using different sampling techniques and adding a new dataset, and classification using a DcCNN. Finally, the models are evaluated using different evaluation metrics and t-distributed stochastic neighbor embedding (t-SNE) plots.

### 3.1. Decorrelated Convolutional Neural Networks

**Figure 2:**
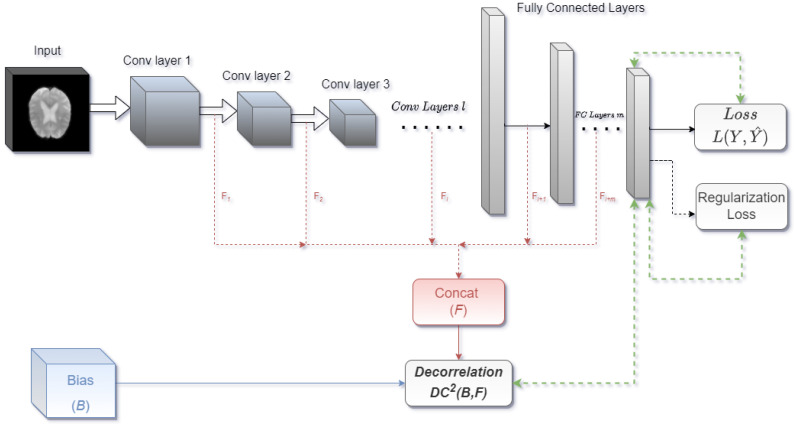
Proposed DcCNN architecture. Red dashed lines denote the output of convolutional layers *l*, which are combined to represent learned features. Green dashed lines indicate the start of the learning process, where backward arrows show back-propagation using their respective gradient values, while forward arrows show forward paths with updated parameters. Network parameters are updated as per the objective function.

Decorrelated convolutional neural networks (DcCNNs) are implemented by applying the decorrelation loss function to CNN architectures. We propose our DcCNN architecture as in Figure 2. We can use one or combinations of any layer outputs and concatenate them as features for the decorrelation function, depending on the complexity of the task. Distance correlation is used as a decorrelation function.

Distance correlation calculates the association between two arbitrary dimension variables using distances. In our proposed approach, $B_{1,...,p}$ is the bias variable. $F_{1,...,p}$ denotes features extracted from the DNN, and *p* is the total number of samples. The distance correlation is the square root of

(1) $$\begin{array}{c} {\mathrm{DC}}^{2}\left(B,F\right)=\left\{\begin{array}{cc} \frac{V^{2}\left(B,F\right)}{\sqrt{V^{2}\left(B,B\right)V^{2}\left(F,F\right)}} & \;\mathrm{if}\;V^{2}\left(B,B\right)V^{2}\left(F,F\right)>0\\ 0 & \;\mathrm{else}\;0 \end{array}\right. \end{array}$$

where DC(B,F) is bounded between 0 and 1. DC(B,F)=0 only if the variables *B* and *F* are independent. $v^{2}\left(B,F\right)$ is the distance covariance between a pair of variables, and $v^{2}\left(B,B\right)$, $v^{2}\left(F,F\right)$ is the distance variance as defined in [36]. The distance covariance is normalized by the distance variances. The Pearson correlation coefficient [37] measures only linear dependencies, but features extracted from a CNN can have nonlinear dependencies, and hence, distance correlation is more preferable since it measures not only linear but also nonlinear dependencies between two random variables.

In our study, we use the squared distance correlation. Class weights are also used in the distance correlation loss function in some of the models to tackle the imbalance problem of scanner data. This function is minimized to reduce the distance correlation between features learned by the networks and the biases. This means that we want to find parameters of the network such that *F* features have a minimal distance correlation with the *B* bias variable. The decorrelation function term is added to the standard objective function for optimization.

### 3.2. Mitigation of Class Bias

Previous research work has shown that imbalanced datasets have a negative impact on the performance of CNNs due to bias towards the majority class. The PPMI dataset used in this study is highly imbalanced, and hence, learning discriminating boundaries between Parkinson’s disease (PD) subjects and healthy control subjects could be more challenging. Our DcCNN models introduce the idea of using the decorrelation loss function along with a data sampling technique to address the class-bias problem in deep learning due to an imbalanced dataset.

#### 3.2.1. PPMI Dataset and Preprocessing

**Figure 3:**
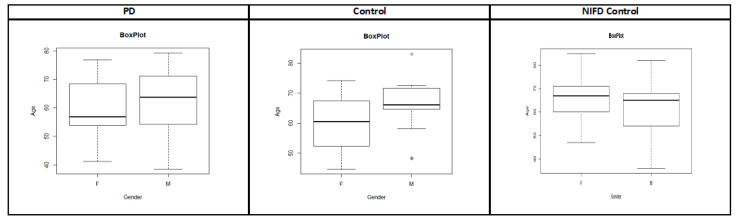
The box plot of age for male and female for PPMI and NIFD datasets.

The PPMI dataset [38] consists of around 183 subjects with follow-up visits. This dataset includes 164 PD patients and 19 healthy control subjects. The demographic information and box plot for the PPMI dataset are shown in Table 1 and Figure 3, respectively. The time required to collect the rs-fMRI data for each subject was around 8 min 4 s. During data collection, subjects were instructed to minimize all movements as well as to rest quietly with eyes open with a clear mind during the scan. They were also instructed to not to fall asleep during this process. For a few subjects, data were collected for up to 1 to 3 years. In this study, imaging data associated with follow-ups are considered independent since they were scanned at different points in time. The size of each rs-fMRI slice is 68 × 66, and these images are in grayscale. A total of 40 axial slices were captured for each subject. The scanner used to collect this dataset is the Tesla scanner manufactured by Siemens Medical Solutions. Functional scans were acquired using the EPI sequence (field strength = 3.0 tesla; flip angle = 80.0 degree; matrix X = 476.0 pixels; matrix Y = 462.0 pixels; Mfg model = TrioTim; pixel spacing X = 3.2941 mm; pixel spacing Y = 3.2941 mm; pulse sequence = EP; volumes = 210.0 time series; slice thickness = 3.2999 mm; TE = 25.0 ms; TR = 2400.0 ms).

The preprocessing of rs-fMRI was performed using FSL v6.0 [39]. An FSL-BET extraction tool [40] was used to extract brain regions and remove skull and neck voxels. Motion correction was performed with the help of the FSL-MCFLIRT toolbox [41] to remove motion artifacts introduced by head movement over time. Spatial smoothing of each volume was implemented using a Gaussian kernel of 5 mm full width at half maximum to reduce noise without reducing the true underlying signal. High-pass temporal filtering with a cut-off frequency of 0.01 HZ (sigma = 90 s) was also applied to remove low-level noise. Since the first ten slices and the last five slices of each subject contain no functional information, they were removed. The end results of this prepossessing for each subject are 66 × 66 PNG images with 25 slices and 210 volumes. The dataset was trained, validated, and tested using 70%, 15%, and 15% of the dataset, respectively. To improve the generalization ability of the DcCNN models, data augmentation methods such as random rotation, random translation, and elastic deformations [42] were applied to the training dataset, which helped to make the model shift, rotation, and deformation invariant.

Since the number of subjects is less and to minimize the overfitting issue, we used each slice and volume as an independent 2D image. Table 2 provides the number of 2D images in the PPMI dataset before oversampling, which clearly indicates an imbalanced dataset since the number of images for PD subjects is more compared to healthy subjects. In order to resolve the class-imbalance problem, different data oversampling techniques such as random oversampling (ROS), the synthetic minority oversampling technique (SMOTE), and stratified sampling were used. ROS [43] is a simple method in which samples from the minority class are randomly increased by making exact copies of existing samples, whereas SMOTE synthetically creates new minority samples by interpolating between minority class samples [44] to balance class distribution. Disproportionate or balanced stratified sampling is a sampling technique that randomly divides the data into different strata in such a way that it samples more data from the minority class samples to balance the samples in the strata [45]. The total number of 2D rs-fMRI images after applying oversampling techniques is shown in Table 2. We also implemented a CNN as a feature extraction technique before applying data sampling methods to evaluate the performance of the models on a class-imbalanced dataset [46]. A simple method, such as the weighted cross-entropy loss function, was also implemented to boost the performance of the DcCNN models by providing more emphasis on the minority class. Our proposed method is a fusion model (oversampling + weighted loss + decorrelation loss), which applies the oversampling technique and includes weighted cross-entropy along with a decorrelation loss function to mitigate class bias.

#### 3.2.2. Decorrelation and Weighted Loss in Objective Function

The models tend to predict most images and subjects as PD patients due to class bias. This class bias is mainly caused by the higher number of PD patients compared to healthy control subjects. In order to represent the class-bias condition quantitatively and to use it as a bias variable in the decorrelation function, we used a dummy bias variable based on discrete uniform distribution. The PD patients group has a wider discrete uniform distribution than the healthy control group, which means the dummy variable will bias the classification results towards PD patients and create class bias. Minimizing the distance correlation between this dummy bias variable and features results in balanced true-positive and true-negative rates.

We introduced the objective function, which consists of three main functions, namely, weighted cross entropy, decorrelation function, and regularizer L2 loss function to mitigate class bias, and is defined as

(2) $$\begin{array}{c} J\left(\theta \right)=\min_{\theta }L_{WCE}\left(Y,\hat{Y}\right)+\lambda {\mathrm{DC}}^{2}\left(B,F\right)+{\left||\theta |\right|}_{2} \end{array}$$

$L_{WCE}$ in Equation (2) represents the weighted binary cross-entropy, and *Y* and $\hat{Y}$ are true and classifier outputs, respectively. The weighted binary cross-entropy simply uses class weights to place more emphasis on the minority class so that the model learns equally from both classes. The decorrelation function is ${DC}^{2}\left(B,F\right)$ where *B* is the dummy class-bias variable and *F* is features extracted from the model. The λ in the objective function is a hyperparameter that determines the relative importance of the decorrelation function in relation to the weighted cross-entropy loss function. The last term ${\left||\theta |\right|}_{2}$ is a regularizer L2 loss function in the objective function for weight decay purposes, which helps to avoid overfitting issues. Optimizing the decorrelation function along with the weighted cross-entropy loss helps to mitigate class bias.

#### 3.2.3. Experimental Setup

The DcCNN model was built by applying decorrelation-based optimization to customized CNN architecture and was trained from scratch. It consists of stacks of three convolutional and max-pooling layers with ReLU activation and a batch normalization layer, two fully connected layers, and softmax as the classifier. These three convolutional layers have 32, 64, and 128 filters, respectively. We used a random oversampling technique to have an equal number of samples between two classes, i.e., PD and healthy control. We used the root means square propagation (RMSprop) optimizer for optimization and weighted cross-entropy and decorrelation function with λ=0.2 as the loss function, as mentioned in Section 3.2.2. A minibatch size of 4000 and an exponential cyclical learning policy [47], which increases and decreases the learning rate by an exponential factor during the training, were used. We observed that an exponential decaying learning rate leads to better generalization. For the decorrelation loss function, we used the outputs of fully connected layers and the softmax layer as features *F*. For the evaluation of the DcCNN model, we used different evaluation metrics such as sensitivity, specificity, precision, and balanced accuracy (BC) calculated from the confusion matrix.

All models in this study were implemented in Python using the TensorFlow platform [48] and cuDNN library [49] on a Linux instance. These experiments were conducted on the AWS Deep Learning AMIs [50] to accelerate deep learning in the cloud using an Amazon EC2 P2 Instance. We used eight high-speed GPUs, parallel processing cores, and single- and double-precision floating-point performance to train the dataset using deep learning. This helped to speed up the training processes. In order to evaluate the performance of all models in this study, the validation dataset was mainly used to determine the optimal values and fine-tune all hyperparameters. It also allowed us to assess the model’s performance during training. To capture uncertainty in the model’s ability in recognizing PD, a 95% prediction interval was calculated on the testing dataset. This was achieved by retraining all models three times and then calculating the mean across these three initializations with a 95% confidence interval.

### 3.3. Mitigation of Scanner Dependencies

A large and balanced neuroimaging dataset is important for deep learning and to improve its generalization ability. Hence, combining all available data from different sites and different scanners plays a vital part in achieving high performance. However, it leads to an increase in variance due to differences in acquisition protocols and scanners. This includes scanner upgrades, scanner manufacturers, scanner strength, etc. We combine PPMI and healthy control subjects from the NIFD dataset to balance the dataset and improve the performance of deep learning to detect PD. The idea behind the proposed DcCNN models is to decorrelate the scanner information and features extracted from models to create scanner-invariant features. Three different variations of the DcCNN model, including DcCNN, feature extraction + DcCNN (FE-DcCNN), which extracts features from the scanner classifier and uses them as the bias variable in DcCNN, and decorrelated convolutional gated recurrent unit DcCNN (ConvGRU-DcCNN), which performs temporal processing, are proposed to mitigate the scanner dependencies.

#### 3.3.1. NIFD Datasets and Preprocessing

We used only rs-fMRI data for healthy controls from the NIFD dataset, and this dataset consists of 215 healthy control subjects with follow-up visits. Just like the PPMI dataset, the demographic information and box plot for the NIFD dataset are shown in Table 1 and Figure 3, respectively. We can see that there is no significant difference in age distribution between the PPMI and NIFD datasets. The size of the rs-fMRI slice is 92 × 92, and the slices are in grayscale. A total of 36 axial slices were captured for each subject. The scanner used to collect this dataset is the Tesla scanner manufactured by Siemens Medical Solutions. Functional scans were acquired using the EPI sequence (field strength = 3.0 tesla; flip angle = 80.0 degree; matrix X = 552.0 pixels; matrix Y = 552.0 pixels; Mfg model = TrioTim; pixel spacing X = 2.5 mm; pixel spacing Y = 2.5 mm; pulse sequence = EP; volumes = 240.0 time series; slice thickness = 3.0 mm; TE = 27.0 ms; TR = 2000.0 ms). As we can see, the scanner manufacturer for the NIFD dataset is the same as the PPMI dataset. However, scanner configurations such as TE, TR, slice thickness, voxel size, and the total number of slices and volumes are different. This might introduce the variance related to scanners, which will ultimately mask the discriminating features between PD and healthy controls. The rs-fMRI data were preprocessed using the same library and steps as the PPMI dataset. Since the first five slices and the last six slices of each subject contain no functional information in the NIFD dataset, they were removed. In order to have the same and fixed size as the PPMI dataset, we also deleted the first 30 volumes in the NIFD dataset. So, the preprocessed NIFD dataset has 66 × 66 PNG images with 25 slices and 210 volumes for each subject. The NIFD dataset is also divided into 70% training, 15% validation, and 15% testing datasets. After combining the PPMI and NIFD datasets, a total of 2,346,750 images were produced, and the class distribution of the combined dataset is provided in Table 3.

#### 3.3.2. Decorrelation in Objective Function

Deep learning models are extremely sensitive to nonbiological variabilities, such as acquisition and scanner settings, in the field of neuroimaging data. One of the important problems in large clinical trials is the scanner dependencies/bias. To deal with the scanner-dependencies issue, we introduced three types of scanner-bias variables, which contain the following: (i) scanner voxel size, i.e., slice thickness and pixel spacing [51], (ii) features extracted from scanner classifier, and (iii) temporal standard deviation to represent scanner-to-scanner variability [31].

The models were trained with a combination of cross-entropy loss $L(Y,\hat{Y})$, the decorrelation loss ${DC}_{control}^{2}\left(B,F\right)$, and the regularizer L2 loss ${\left||\theta |\right|}_{2}$ functions. This objective function can be expressed as

(3) $$\begin{array}{c} J\left(\theta \right)=\min_{\theta }\lambda_{1}L(Y,\hat{Y})+\lambda_{2}{\mathrm{DC}}_{control}^{2}\left(B,F\right)+{\left||\theta |\right|}_{2} \end{array}$$

where *L* is the softmax cross-entropy loss and ${\left||\theta |\right|}_{2}$ is the regularizer L2 loss function. The decorrelation function is ${DC}_{control}^{2}\left(B,F\right)$, where *B* is the scanner-bias variable, *F* is features extracted from the model, and subscript control indicates that the decorrelation function is only applied to control subjects since the healthy control subjects were scanned using both the scanners with different acquisition protocols, i.e., present in PPMI as well as NIFD datasets, whereas PD subjects were scanned using only one scanner out of two scanners, i.e., present in only the PPMI dataset. This helps the models to remove scanner-related information rather than removing the main task, i.e., PD detection-related information. $\lambda_{1}$ and $\lambda_{2}$ in the objective function are hyperparameters that control the trade-off between the cross-entropy loss function and the decorrelation function. Since the number of healthy controls in the PPMI dataset is less compared to the NIFD dataset, higher class weights were assigned to PPMI controls than NIFD controls to make decorrelation loss for PPMI controls larger than NIFD controls. This helps the models to decorrelate features equally from both scanners and ultimately to resolve imbalanced scanner data problems for healthy controls.

#### 3.3.3. Experimental Setup

We trained three different DcCNN models with different architectures and scanner-bias variables. The first model, abbreviated as DcCNN, has the same architecture as the DcCNN model used to mitigate class bias except for changes in the objective function to mitigate scanner dependencies, and there are three stack convolutional layers with 32, 16, and 16 filters, followed by two hidden layers with 40 and 100 neurons. We trained DcCNN with a minibatch size of 4000 and an exponential cyclical leaning policy using an RMSProp optimizer for optimization with a decay of 0.005. Hyperparameters $\lambda_{1}=0.5$ for cross-entropy loss and $\lambda_{2}=5.0$ for decorrelation function were used to control the trade-off between two loss functions as mentioned in Section 3.3.2. The output of the first convolutional layer and fully connected layers are used as feature *F*, while slice thickness and pixel spacing are considered as scanner information and used as scanner-bias variable *B*.

**Figure 4:**
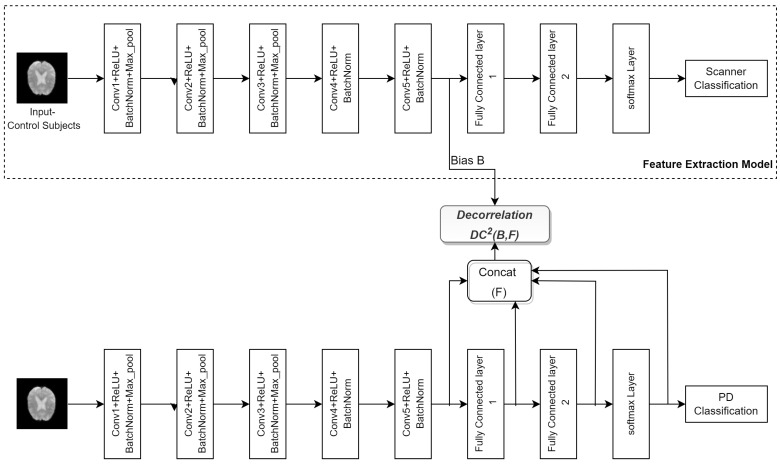
The architecture of the FE-DcCNN model.

The second model (FE-DcCNN) comprises two models. The first model was built to predict the scanner, and we refer to it as the feature extraction (FE) model. The dataset used to train this model consists of only healthy control subjects from the PPMI and NIFD datasets. Once the training is performed, features are extracted from the FE model and used as a scanner-bias variable in the second model, i.e., the DcCNN model. Both FE and DcCNN models have the same architecture and the same training dataset. These models have five stacks of convolution, batch normalization, and max-pooling layers with ReLU activation, as shown in Figure 4, followed by two fully connected layers with 40 and 100 neurons. Both models use 32, 16, 16, 8, and 8 filters to extract discriminative features for the detection of PD. The output of the fifth convolutional layer in the FE model is used as scanner-bias variable *B*, whereas the outputs of the fifth convolutional layer, along with fully connected layers in the DcCNN model, are used as feature *F*. The hyperparameters used in the objective function are $\lambda_{1}=0.05$ and $\lambda_{2}=0.95$. We used a dropout of 0.2 in the first four convolutional layers to reduce the overfitting problem in the model, and the rest of the training configuration is the same as the first model, DcCNN.

**Figure 5:**
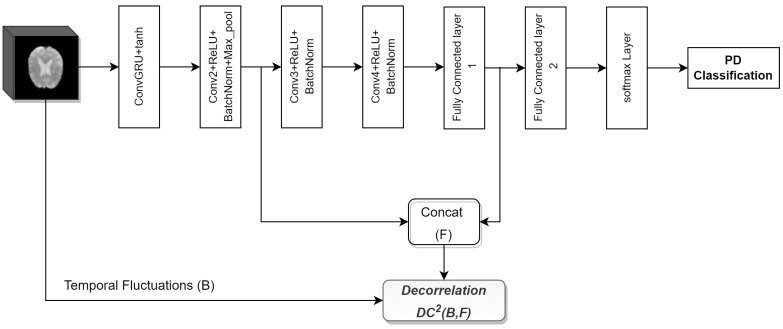
The architecture of the ConvGRU-DcCNN model.

To make use of temporal information present in rs-fMRI, we implemented the third model (ConvGRU-DcCNN) as shown in Figure 5. ConvGRU-DcCNN performs temporal processing first and uses a 3D image of size 66 × 66 × 210 as the input. Since we have to use temporal information for this model, we converted 2D images to 3D images, which produced a total of 11,175 images, including 5450 PD and 5725 healthy control PNG samples. The core architecture consists of convolutional gated recurrent operations (convGRU) [52] as the first layer, followed by the DcCNN architecture. ConvGRU is used to perform temporal processing. The DcCNN part consists of three convolutional layers with filters 16, 32, and 32, followed by two fully connected layers with 1000 and 500 neurons. The model was trained using an Adam optimizer with a minibatch size of 256 and a learning rate (lr) scheduler with an initial lr of 0.001 with a decay of 0.5. In addition to this, an optimizer weight decay of 0.005 was used. We used $\lambda_{1}=0.2$ and $\lambda_{2}=0.6$ in the objective function. For decorrrelation loss, we used the output of the second convolutional layer and the first fully connected layer as features *F*, while temporal standard deviation (temporal fluctuations) was used as scanner-bias *B*.

## 4. Results

In order to assess whether the DcCNN models performed better to mitigate class bias and scanner bias, we applied our method and baseline model to the single-scanner imbalanced PPMI dataset and the combination of multiscanner PPMI and NIFD datasets, respectively.

### 4.1. Single-Scanner Imbalanced Dataset

**Figure 6:**
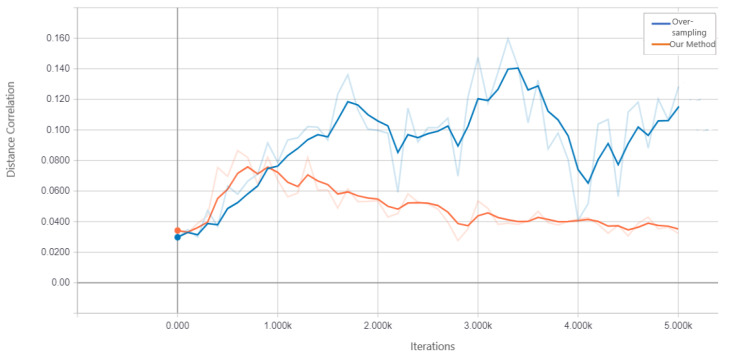
Distance correlation between learned features and class bias for the imbalanced dataset.

We assess the performance of DcCNN to classify PD on the PPMI imbalanced dataset. Our proposed fusion method aims to mitigate class bias. In order to show that DcCNN reduces the statistical dependence between features and class bias variables, we plot the distance correlation against iterations as shown in Figure 6. The plot shows that distance correlation decreases as the iteration increases for our fusion model as opposed to the oversampling method. We compare our fusion model with different CNN models and existing data-sampling techniques. The baseline model is a simple CNN model and has the same architecture as DcCNN, where no data-sampling techniques and class-bias mitigation methods are applied. The existing data-sampling techniques, ref. [53] such as smote and oversampling, are implemented to address the class-imbalance issue. We also compared our model with a fusion of different combinations of existing class-bias mitigation techniques, such as a fusion of oversampling and weighted loss functions, a fusion of feature extraction (FE) and smote, and stratified sampling.

**Figure 7:**
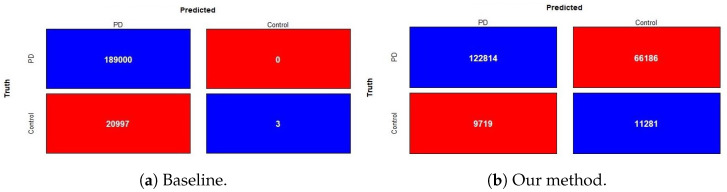
Confusion matrix of baseline and our method (ROS + weighted loss + DcCNN) with two classes for imbalanced PPMI testing dataset (slice-level PD recognition).

**Figure 8:**
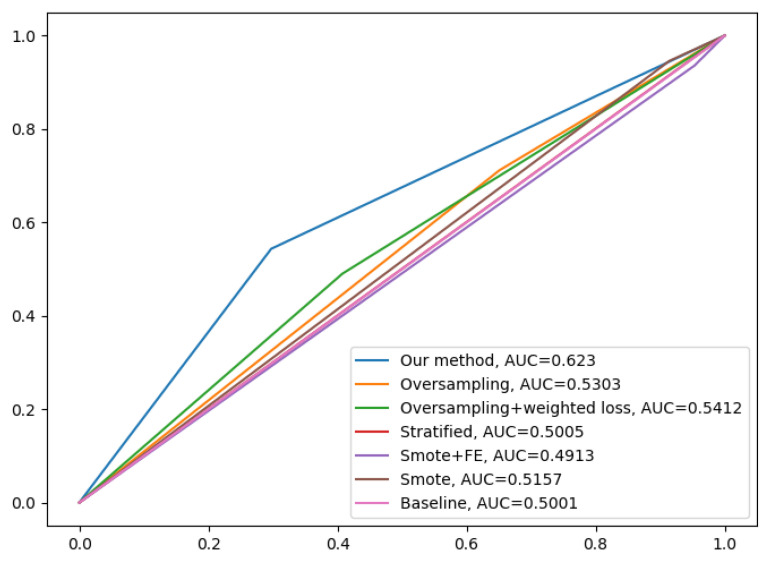
ROC curves of different methods for imbalanced dataset. The X axis represents the false-positive rate, and Y axis represents the true-positive rate.

The results of the holdout testing dataset for each method are displayed in Table 4, and the performance of imbalanced classification is measured specifically by sensitivity, specificity, precision, and balanced accuracy (BA). As we can see from the results, our proposed fusion method significantly increases balanced accuracy as compared to other methods. This, therefore, suggests that using the decorrelation function along with the oversampling technique and weighted loss function creates features that are invariant to class bias. The precision and specificity are higher for our fusion model compared to other methods. Lower sensitivity and higher specificity for our fusion model indicate that model prediction is not biased towards the majority class, i.e., PD subjects, whereas higher sensitivity and lower specificity for methods such as baseline, smote, FE+smote, stratified sampling, and oversampling indicate model prediction is highly biased towards the PD class. The lower values of specificity for these models clearly demonstrates that the classification of control subjects are almost based on random chance. For all these existing models, we notice that the weighted loss function helps the model to improve balanced accuracy. Figure 7 shows the confusion matrix of the baseline model and our proposed DcCNN model to classify slices into the PD and healthy control categories. The confusion matrix for the baseline model clearly indicates that all subjects are classified as PD due to the presence of class bias, while our proposed model classifies both classes almost equally by mitigating this class bias. Figure 8 illustrates the ROC curve of different methods. From this graph, we observe the superior performance of our fusion DcCNN model over traditional data-sampling methods. In both balanced accuracy and ROC metrics, our DcCNN fusion method clearly outperforms other methods.

Due to the few labeled rs-fMRI images available at the subject level in the PPMI dataset, we trained the models at the slice level, which increases the data and avoids the overfitting issue. The above-reported results are for the slice-level classification. Since in the medical field, subject-level PD classification is important, we propose a global subject-level classification by using a max-wins voting strategy. In this strategy, all slices for each subject are classified, and then the class with the maximum votes for a given subject determines the global subject classification. This allows the model to classify and assign PD or healthy control labels to a given subject. As shown in Table 5, applying the max-wins voting strategy for subject-level classification significantly improved accuracy by correcting a small number of misclassified slices. Our fusion DcCNN model achieves a subject-level balanced accuracy of 67% after applying a max-wins voting strategy.

### 4.2. Multiscanner Datasets

**Figure 9:**
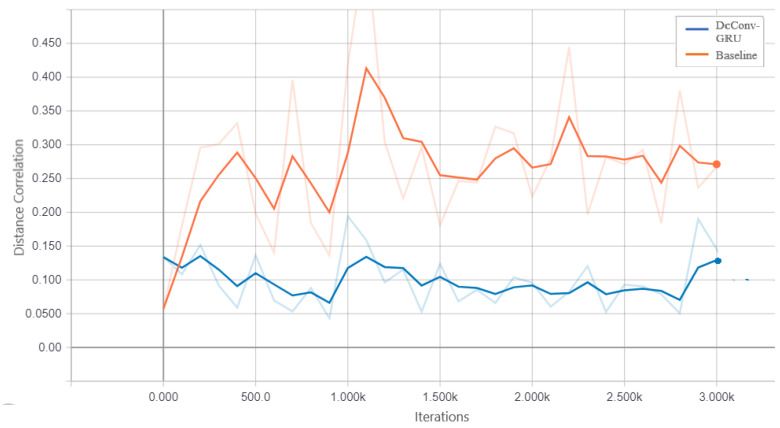
Decorrelation between learned features and scanner bias for baseline ConvGRU-CNN and ConvGRU-DcCNN models.

The DcCNN, FE-DcCNN, and ConvGRU-DcCNN modelsare the three main models presented in this subsection to create features that are invariant to scanner and acquisition protocols while maintaining the performance of PD classification. This will reduce the influence of the scanner on model predictions. We compare our proposed models with baseline models. In a similar way to the previous imbalanced dataset experiment, baseline models such as CNN and ConvGRU-CNN share the same architecture as DcCNN and ConvGRU-DcCNN, respectively, without any scanner-bias mitigation methods being incorporated. Figure 9 shows that statistical dependence between learned features and scanner bias decreases as iteration increases for ConvGRU-DcCNN as opposed to the baseline ConvGRU-CNN model. The purpose of this plot is to observe the trend rather than to show the true difference between the distance correlation values of the ConvGRU-DcCNN model and the baseline model since weights were assigned to calculate the decorrelation function used in ConvGRU-DcCNN versus the baseline model. We also evaluate the performance of scanner bias mitigation techniques using accuracy, scanner classification accuracy, and error rate for each dataset/scanner (since each dataset represents one scanner). The scanner classification accuracy indicates the scanner information present in features that influence the decision of model prediction.

Table 6 presents the performance of different types of DcCNN models on a multiscanner testing dataset. As expected, the scanner classification accuracy for baseline models is 100% which means models make predictions based on features that are dependent on the scanner and not on the main task of PD recognition. With the FE model, the scanner classification was performed using only healthy control groups, and scanner-relevant features were extracted for the FE-DcCNN model to use as scanner-bias variables. The FE model results in an accuracy of 92.6% at the slice level and 100% at the subject level. All three types of DcCNN models reduce the scanner classification accuracy compared to baseline models, indicating that DcCNN reduces scanner dependencies fairly with a slight reduction in accuracy. Accuracy for baseline models is high due to the fact that all PD subjects in the dataset were scanned on one scanner, and the majority of the healthy control subjects were scanned on another scanner. Thus, it makes the task harder, and we can see a reduction in accuracy for DcCNN when compared with baseline models. Hence, for our multiscanner dataset, we can say that raw classification accuracy is not only a consideration. The error rates for both datasets (i.e., both scanners) increase for DcCNN models, indicating that scanner bias removal is performed. The convGRU-DcCNN model has poorer performance compared to the DcCNN and FE-DcCNN models in terms of accuracy, possibly because it removes information related to the main task while reducing scanner dependencies. The ConvGRU-DcCNN model performs poorly, most likely due to four factors: removal of PD-relevant features, decorrelation penalization leading to a negative influence on predictive accuracy, reduction in data size, and inclusion of PD information in scanner bias variable. DcCNN and FE-DcCNN models have similar accuracy while substantially decreasing the scanner dependencies.

**Figure 10:**
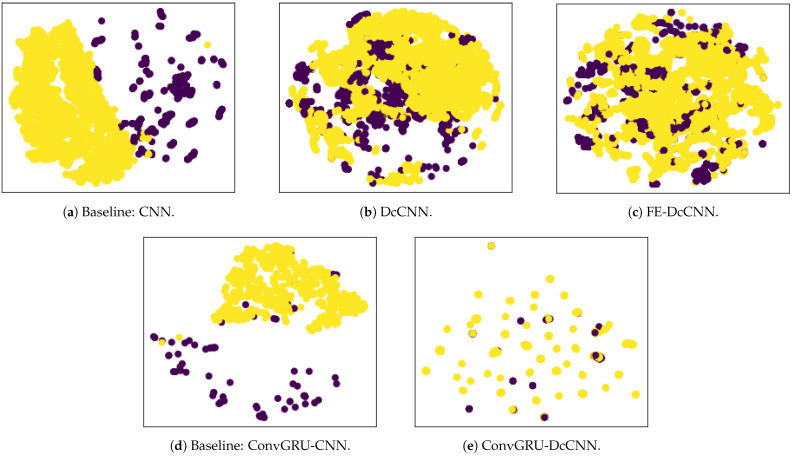
tSNE plot of the learned fully connected layer features for healthy control data. The yellow color indicates the NIFD dataset scanner, and the purple color indicates the PPMI dataset scanner.

Finally, the above results are further supported by t-distributed stochastic neighbor embedding (t-SNE) visualizations of the learned fully connected layer features, as shown in Figure 10. Since only healthy control subjects were scanned using both scanners and are present in both datasets, we plot tSNE visualization for the healthy control group. We observe that the baseline models, such as CNN and ConvGRU-CNN, have a clear association with the scanner since the PPMI dataset is grouped on the right side, while the NIFD dataset is grouped on the left side of Figure 10a. However, scanner features become jointly embedded in DcCNN, FE-DcCNN, and ConvGRU-DcCNN models, which indicate no apparent bias towards the scanner. This suggests that our proposed DcCNN models successfully create features that are invariant with respect to scanners without compromising the performance of PD classification. For the FE-DcCNN model, data points in Figure 10b are largely indistinguishable across both scanners compared to the DcCNN model in Figure 10c. This can also be confirmed because the scanner classification accuracy for FE-DcCNN is lower than for the DcCNN model. Similar to FE-DcCNN, the features learned by the ConvGRU-DcCNN model spread uniformly across all scanners, indicating successful mitigation of scanner dependencies, but the ConvGRU-DcCNN model results in a drastic loss of accuracy, indicating the removal of information related to the main task.

For subject-level classification, we used the same max-wins voting strategy as defined for a single-scanner imbalanced dataset. The above-reported results for multiscanner datasets are for the subject-level classification. The evaluation metrics for slice-level and subject-level classification are summarized in Table 7. All these results show that the FE-DcCNN model not only successfully mitigates the scanner bias but also achieves high performance in comparison with DcCNN and ConvGRU-DcCNN models, respectively. The FE-DcCNN model achieves a subject-level accuracy of 78% after applying a max-wins voting strategy and scanner classification accuracy of 80%.

## 5. Discussion

This study presents a decorrelation-based bias mitigation technique that can be applied to deep learning architectures such as CNN, ConvGRU, and fusion methods to mitigate not only class bias but also scanner bias by creating class- and scanner-invariant features. We demonstrated that our decorrelation technique can be applied to any architecture and provides a high level of flexibility. The hyperparameter λ>0 plays a vital role in deciding the importance of decorrelation and regular loss function. When λ=0, it means it is a baseline model with no bias mitigation technique applied. Extreme high values of λ will cause unstable training and poor classification performance. Hence, finding optimal values for hyperparameters λ is crucial and can be achieved by trying different values of λ. The values and range for λ vary for different models depending upon network architecture and the complexity of the mitigation task. We notice that increasing the batch size improves the stability of the decorrelation function during training. In addition, it provides unbiased estimates of distance covariance when the batch size is larger. Similar to hyperparameter λ, we found that finding the optimal combination of the output of layers as feature *F* helps in improving the performance of the bias mitigation technique. The choice of feature *F* depends on the type of bias mitigation technique and model architecture. As stated in our previous work [35], the bias variable *B* should provide more precise bias-relevant information.

The original rs-fMRI imaging data are organized in 4D matrices, which contain spatial as well as temporal information. Due to high dimensionality and small dataset size, deep learning models face problems like overfitting when 4D data are used. This would only be solved by adding more data. However, 2D and 3D rs-fMRI data used in this study show the applicability of using these data for PD classification while significantly mitigating the class and scanner biases. We also find that the ConvGRU-DcCNN model almost exhibits similar performance with and without class weights for the decorrelation function since using temporal information reduces the size of the dataset and, ultimately, the imbalance ratio between PPMI controls and NIFD controls. Out of the three types of scanner bias variables used to mitigate scanner bias, features extracted from the scanner classifier bias variable provide more accurate scanner-relevant information since the FE-DcCNN model yields optimal results, which reduces scanner dependence without removing much PD-specific information.

The results from the class bias mitigation study show that we were able not only to achieve higher performance than existing traditional approaches but also to successfully mitigate bias towards the majority class. We also showed that the same decorrelation function technique can be used to remove scanner dependencies. The scanner classification accuracy and tSNE plots confirm that scanner dependencies were reduced. Since existing harmonization and domain adaptation methods approach scanner mitigation differently than our method, we do not directly compare them to our method. Additionally, our proposed model differs from previous methods in that it is designed for rs-fMRI data collected from a single scanner with identical acquisition protocols and a single site rather than from multiscanner and multisite data. Furthermore, the existing deep learning approaches, such as LSTM and attention networks, focus only on using time series data, which require the extraction of ROIs and prior knowledge regarding PD-specific ROIs, whereas our proposed method neither requires extraction of ROIs nor loses the spatial information present in the rs-fMRI data. The presented method also suggests that combining multiscanner data and increasing the size of the dataset improves the performance of PD classification compared to single-scanner imbalanced data. This further indicates that increasing the size of the dataset by combining multiscanner data enhances the model generalization ability of the presented method, as opposed to utilizing data-sampling techniques with single-scanner data.

The comparison results with the existing model highlight the deterioration in the performance of the existing deep learning model due to the current bias problems present in the neuroimaging domain, which ultimately affects the reliability and implies the need for bias-invariant methodology, especially in the medical field. The findings of this study demonstrate the effectiveness of the new proposed model in recognizing PD with accurate and reliable model outcomes, even in the presence of bias. Therefore, this research study may be of further use to the medical community in a screening setting for diagnosing PD and finding solutions to stop or avoid the progression of the disease. This study contributes to the field by resolving the issue of deep learning models making erroneous decisions due to biases present in the neuroimaging data. This issue has not been thoroughly explored in the literature. Our findings not only highlight the importance of understanding how class and scanner biases impact the ability of deep learning in decision making but also present an effective and straightforward decorrelation-based deep learning approach to mitigate any irrelevant dependencies, such as class and scanner biases.

## 6. Conclusions

The performance of deep learning models is highly impacted by bias variability and class imbalance present in data. We introduce a novel decorrelation approach, which reduces the distance correlation between the features learned by deep learning models and biases. The main goal of this approach is to mitigate scanner dependencies and class bias, which will help the model to generalize to multiscanner and multicenter datasets. The proposed framework includes extensive data preprocessing modules and decorrelated deep learning-based classifiers to distinguish PD patients from healthy controls using rs-fMRI data. We evaluated our four different models on single-scanner imbalanced and multiscanner datasets. On a single-scanner imbalanced PPMI dataset, our proposed DcCNN model significantly improves performance by alleviating bias toward the majority class, whereas our proposed FE-DcCNN model produces scanner-invariant features without affecting accuracy much on multiscanner PPMI and NIFD datasets. Furthermore, the rs-fMRI dataset is used for the first time to train CNN models for PD classification. These simple yet efficient proposed DcCNN models perform better than previous approaches and baseline models to mitigate the bias and require fewer hyperparameters to optimize. We additionally verify from the results that using a multiscanner and larger dataset results in significantly better performance when compared with a single-scanner imbalanced dataset. This study also demonstrated that subject-level classification results in an even more robust model and improves accuracy using a max-wins voting strategy.

An immediate next step would be using advanced visualization techniques such as saliency maps, DeepLIFT, and occlusion maps. A combination of these precise and detail-oriented visualization techniques may help in characterizing fMRI biomarkers for PD. Our proposed models also demonstrate the potential for predicting stages in the progression of PD, which could be addressed in future studies. Additional future directions of our work also include collecting a larger dataset and more information related to patients along with individual rs-fMRI slices and temporal information to achieve higher accuracy and reliability. A larger dataset and increased computation complexity will also enhance the overall performance of 4D-DcCNN models by taking advantage of using the inherent spatial–temporal information in 4D rs-fMRI data. Moreover, it would be interesting to investigate how the application of the proposed decorrelation approach to pretrained models and to different types of data variations and biases would impact performance.

## Figures and Tables

**Table 1:** Demographic information of two datasets, PPMI and NIFD.

Datasets	Total Subjects	Group	Subjects	Gender	Subjects	Mean of Age	SD of Age	Min of Age	Max of Age
PPMI	183	PD	164	Male	111	62.55	10.53	38.6	79.3
				Female	53	59.65	9.36	41.2	76.9
		Control	19	Male	15	65.98	9.04	48.1	83.1
				Female	4	59.95	12.1	44.6	74.2
NIFD	215	Control	215	Male	129	62.6	8.45	36	82
				Female	86	65.9	7.95	47	85

**Table 2:** Class distribution of PPMI training dataset before and after oversampling.

Class	Number of Images	
	**Before Oversampling**	**After Oversampling**
PD	818,790	818,790
Control	90,930	818,790

**Table 3:** Class distribution of combined PPMI and NIFD datasets.

Class	Number of Images		
	**Training**	**Validation**	**Testing**
PD	813,750	141,750	189,000
Control	819,000	178,500	204,750

**Table 4:** Performance evaluation of PD classification for imbalanced PPMI dataset using different methods.

Methods	Sensitivity	Specificity	Precision	BA
Baseline	100.00%	0.01%	90.00%	50.01%
Smote	94.60%	8.60%	90.30%	51.60%
FE + Smote	93.60%	4.70%	89.80%	49.15%
Stratified	95.60%	4.50%	90.00%	50.05%
Oversampling	71.20%	34.90%	90.80%	53.05%
Oversampling + weighted loss	49.00%	59.20%	91.50%	54.10%
Our method	58.47%	60.37%	93.07%	59.42%

**Table 5:** Sensitivity (%), specificity (%), precision (%), and balanced accuracies (%) of slicewise and subjectwise PD recognition for imbalanced PPMI testing dataset. Results are mean across three initializations with a 95% confidence interval.

Methods	Sensitivity	Specificity	Precision	BA
Slice-level	58.47 ± 0.05	60.37 ± 0.08	93.07 ± 0.01	59.42 ± 0.03
Subject-level	66.67 ± 0.08	66.67 ± 0.20	95.13 ± 0.03	66.67 ± 0.10

**Table 6:** Performance evaluation of baseline models and DcCNN models using PPMI and NIFD datasets.

Models	Accuracy	Scanner Classification Accuracy	NIFD Error Rate	PPMI Error Rate
Baseline models:				
CNN	94.70%	100.00%	0.00	0.00
ConvGRU-CNN	94.70%	100.00%	0.00	0.00
Our models:				
DcCNN	80.47%	83.10%	0.25	0.06
FE-DcCNN	77.80%	80.43%	0.30	0.17
ConvGRU-DcCNN	65.77%	63.13%	0.46	0.28

**Table 7:** Sensitivity (%), specificity (%), precision (%), F1 (%), and accuracies (%) of slicewise and subjectwise PD recognition for PPMI and NIFD testing datasets. Results are mean across three initializations with a 95% confidence interval.

Models	Methods	Sensitivity	Specificity	Precision	F1	Accuracy
DcCNN	Slicewise	76.87 + 0.06	78.00 ± 0.08	76.90 ± 0.05	76.60 ± 0.01	77.47 ± 0.02
	Subjectwise	79.63 + 0.09	81.20 ± 0.10	80.43 ± 0.06	79.57 ± 0.03	80.47 ± 0.03
FE-DcCNN	Slicewise	83.40 + 0.14	71.00 ± 0.06	72.70 ± 0.02	77.20 ± 0.05	76.95 ± 0.03
	Subjectwise	80.53 + 0.16	75.20 ± 0.05	75.03 ± 0.01	77.13 ± 0.07	77.80 ± 0.05
ConvGRU-DcCNN	Slicewise/ Subjectwise	74.07 + 0.03	58.13 ± 0.01	62.00 ± 0.002	67.50 ± 0.01	65.77 ± 0.01

## Data Availability

Not applicable.

## Abbreviations

The following abbreviations are used in this manuscript:

PD	Parkinson’s disease
rs-fMRI	Resting-state functional MRI
CNN	Convolutional neural network
DcCNN	Decorrelated convolutional neural network
PPMI	Parkinson’s Progression Markers Initiative
NIFD	FTLDNI—frontotemporal lobar degeneration neuroimaging initiative
t-SNE	t-distributed stochastic neighbor embedding
t-SNE	t-distributed stochastic neighbor embedding
FE-DcCNN	Feature extraction-DcCNN
